# Machine learning-based warning model for chronic kidney disease in individuals over 40 years old in underprivileged areas, Shanxi Province

**DOI:** 10.3389/fmed.2022.930541

**Published:** 2023-01-09

**Authors:** Wenzhu Song, Yanfeng Liu, Lixia Qiu, Jianbo Qing, Aizhong Li, Yan Zhao, Yafeng Li, Rongshan Li, Xiaoshuang Zhou

**Affiliations:** ^1^School of Public Health, Shanxi Medical University, Taiyuan, Shanxi, China; ^2^Department of Nephrology, Shanxi Provincial People’s Hospital (Fifth Hospital) of Shanxi Medical University, Taiyuan, China; ^3^Shanxi Provincial Key Laboratory of Kidney Disease, Taiyuan, China; ^4^Core Laboratory, Shanxi Provincial People’s Hospital (Fifth Hospital) of Shanxi Medical University, Taiyuan, China; ^5^Academy of Microbial Ecology, Shanxi Medical University, Taiyuan, China

**Keywords:** machine learning, chronic kidney disease, albuminuria-to-creatinine ratio, α1-microglobulin-to-creatinine ratio, auxiliary diagnosis, warning model

## Abstract

**Introduction:**

Chronic kidney disease (CKD) is a progressive disease with high incidence but early imperceptible symptoms. Since China’s rural areas are subject to inadequate medical check-ups and single disease screening programme, it could easily translate into end-stage renal failure. This study aimed to construct an early warning model for CKD tailored to impoverished areas by employing machine learning (ML) algorithms with easily accessible parameters from ten rural areas in Shanxi Province, thereby, promoting a forward shift of treatment time and improving patients’ quality of life.

**Methods:**

From April to November 2019, CKD opportunistic screening was carried out in 10 rural areas in Shanxi Province. First, general information, physical examination data, blood and urine specimens were collected from 13,550 subjects. Afterward, feature selection of explanatory variables was performed using LASSO regression, and target datasets were balanced using the SMOTE (synthetic minority over-sampling technique) algorithm, i.e., albuminuria-to-creatinine ratio (ACR) and α1-microglobulin-to-creatinine ratio (MCR). Next, Bagging, Random Forest (RF) and eXtreme Gradient Boosting (XGBoost) were employed for classification of ACR outcomes and MCR outcomes, respectively.

**Results:**

12,330 rural residents were included in this study, with 20 explanatory variables. The cases with increased ACR and increased MCR represented 1,587 (12.8%) and 1,456 (11.8%), respectively. After conducting LASSO, 14 and 15 explanatory variables remained in these two datasets, respectively. Bagging, RF, and XGBoost performed well in classification, with the AUC reaching 0.74, 0.87, 0.87, 0.89 for ACR outcomes and 0.75, 0.88, 0.89, 0.90 for MCR outcomes. The five variables contributing most to the classification of ACR outcomes and MCR outcomes constituted SBP, TG, TC, and Hcy, DBP and age, TG, SBP, Hcy and FPG, respectively. Overall, the machine learning algorithms could emerge as a warning model for CKD.

**Conclusion:**

ML algorithms in conjunction with rural accessible indexes boast good performance in classification, which allows for an early warning model for CKD. This model could help achieve large-scale population screening for CKD in poverty-stricken areas and should be promoted to improve the quality of life and reduce the mortality rate.

## Introduction

Chronic kidney disease (CKD) is defined as renal structural or functional abnormalities for 3 months, with a prevalence of 13.4% worldwide (1). In recent years, CKD has gradually moved away from the perception of “disease of the elderly,” with a growing trend toward age groups. Yet, imperceptible symptoms at the initial stages and lower public awareness are often responsible for the missing of golden treatment time (2). More seriously, it may develop into end-stage renal disease that requires renal replacement therapy, leading to a decrease in quality of life and an increase in mortality. What’s worse, it’s highly related to complications such as cardiovascular disease (3), becoming another “silent killer” after tumours and diabetes. As such, its early screening enables patients and their families to plan ahead, consult professional doctors for treatment, and discuss lifestyle modifications, which contribute to the alleviation of CKD.

Accurate diagnosis is closely related to the detection of CKD. Undoubtedly, albuminuria and α1-microglobulin serve as a good approach for early CKD screening in tertiary hospitals. Yet, when it comes to the rural areas in China, health care workers, critical care units, emergency facilities, health services and medical examinations are not necessarily guaranteed (4–6). In such a situation, it is not practical for primary health care institutions to make an early CKD screening using urine protein (7). Therefore, it is worth considering how to maximise the accessible medical parameters to construct a warning model for CKD in rural areas under the existing health care conditions.

Previous studies demonstrated that some risk factors, such as demographic, lifestyle, and blood biochemical parameters could be used to predict disease occurrence (8, 9). Also, it has been documented that the machine-learning approach allows for higher accuracy of disease risk prediction using routine clinical data, facilitating better decision support for clinicians (10). Accordingly, it is of great significance to combine machine learning algorithms with those easily accessible medical indexes to construct a warning model for CKD in poverty-stricken areas.

In this study, we aimed to employ machine learning algorithms for early screening of CKD and developed a warning model targeted at poverty-stricken areas using demographic, blood biochemical and physical examinations data from ten rural areas in Shanxi Province, intending to achieve a larger-scale CKD screening programme at a lower cost (reduced financial expenditure, reduced burden on medical staff) where possible, thus shifting forward its treatment window and improving quality of life.

## Participants and methods

### Study participants

From April 2019 to November 2019, Shanxi Provincial People’s Hospital carried out opportunistic screening for chronic kidney disease for residents over 40 years old in Ningwu County, Lu County, Yangqu County, Linxian County, Shouyang County, Zezhou County, Huozhou City, Hejin City, Linyi County and Ruicheng County in Shanxi Province. Up to 13,550 residents participated in this screening and 12,285 eventually enrolled in the study, including 5,206 men and 7,079 women aged 41–91 years. All study subjects signed informed consent forms and it was approved by the Shanxi Provincial Hospital Ethics Committee (No. 2021213).

Inclusion criteria: (1) Residents aged 40 years and above; (2) Participants without communication barriers; (3) Participants understanding the study significance and willing to sign a written informed consent; (4) participants without cognitive impairment or mental illness. Exclusion criteria: (1) incomplete information recorded; (2) poor compliance; (3) pregnant women or those with a history of drug abuse.

### Data collection

Data is collected through questionnaires, physical examinations, and laboratory analysis. The questionnaire included demographic information (including age, sex, annual income, educational levels), lifestyle (including smoking, alcohol consumption, diet and exercise). Questionnaires were conducted online and were completed by the subjects themselves or their family members. Physical examination comprised height, weight and systolic blood pressure (SBP), diastolic blood pressure (DBP), measured twice and averaged. All data were measured by medical professionals. Body mass index (BMI) was calculated by weight in kilograms divided by the square of height in meters.

Fasting venous blood was collected from subjects for fasting blood glucose (FPG), glycated haemoglobin (GHb), homocysteine (Hcy), total cholesterol (TC), triglycerides (TG), low-density lipoprotein cholesterol (LDL-C) and high-density lipoprotein (HDL-C). Urine specimens were collected from subjects. After centrifugation of 3,000 r/min for 10 min, the supernatant (low-speed centrifuge Anhui Zhongke Zhongke A SC3616) was extracted, and latex turbidity, sarcosine oxidase and immunoturbidimetry were employed for detection of α_1_-microglobulin (α_1_MG), urine creatinine (UCr) and microalbuminuria (MAU), respectively.

### Variable assignments

Study participants’ annual income, educational levels, health history, and lifestyle information was obtained from the questionnaire. Annual income was defined as <5K yuan, 5–10K yuan, 10–20K yuan, >20K yuan; educational attainment was defined as ≤ primary school, ≤ middle school, ≤ high school, ≥ college; smoking was classified as yes or no; alcohol consumption was classified as always (>100 g/time and 3 times/week), sometimes (<3 times/week or < 100 g/time) and rarely; exercise was classified as “none or a little” or “regular” (≥3 times/week, ≥30 min/time). BMI was defined as underweight (<18.5 kg/m^2^), normal (18.5–24.0 kg/m^2^), overweight (24.0–28.0 kg/m^2^), obesity (≥28 kg/m^2^). ACR was defined as MAU divided by UCr multiplied by 8.84; MCR was defined as α_1_MG divided by UCr multiplied by 8.84.

Explanatory variables include demographic information (age, sex, educational levels, annual income, residence), lifestyle (smoking, alcohol, exercise, salt consumption, diet), blood biochemistry (HDL, LDL, TG, TC, Hcy, FPG, GHb), physical examination indexes (SBP, DBP, BMI), a total of 20 variables. The response variables were defined as ACR outcomes or MCR outcomes with two classes (increased ACR or normal ACR and increased MCR or normal MCR) ACR ≥ 30 mg/g was defined as increased ACR; MCR > 23 mg/g was defined as increased MCR. The increased ones were assigned 1, and the normal ones were assigned as 0.

### Data preprocessing

**FIGURE 1:**
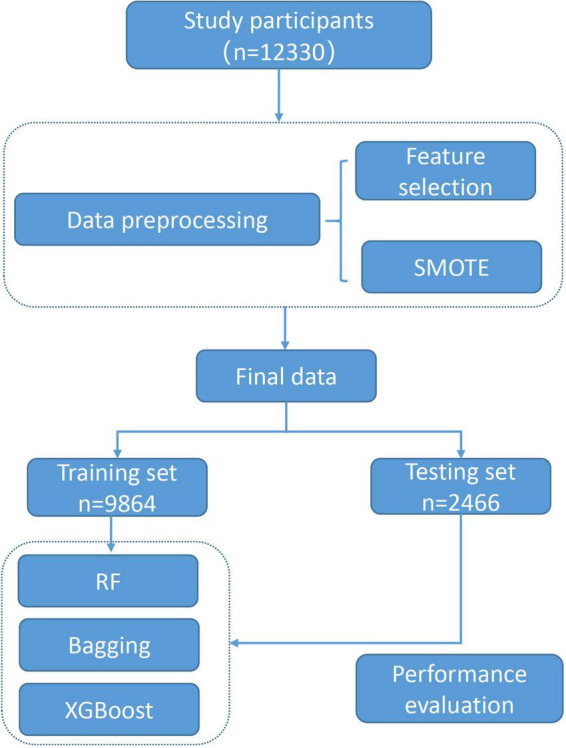
Workflow of the model construction.

Since missing data is not an issue of great severity in this study, we excluded those samples with missing values, without making a data imputation. Then, Absolute Shrinkage and Selection Operator (LASSO) was used to select features that are more relevant to the response variables, the increased ACR and the increased MCR. Afterward, Synthetic Minority Over-Sampling Technique (SMOTE) was utilized to balance the classes to enable the machine learning models to better learn the data features, thus making the best classification. The workflow was shown in Figure 1.

LASSO represents one of the commonly used feature selection methods. It is characterized by adding L1 regularization penalty term when fitting generalized linear regression, so that the sum of absolute values of regression coefficients is less than a certain value. Its purpose is to minimize the sum of squares of residuals, and force the regression coefficients of variables that contribute less to the model to compress to zero, enabling a feature sparsity process (11, 12). It could eliminate predictors with autocorrelation or redundancy, allowing for automated variable selection within the model, and significantly contributing to the performance of classification models (13, 14).

**FIGURE 2:**
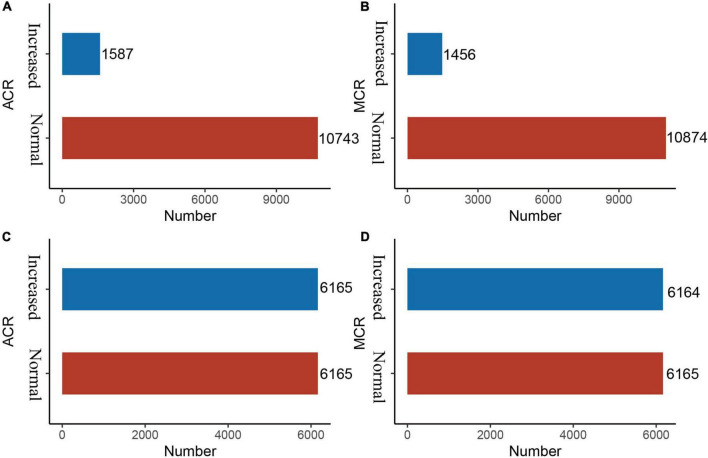
Before and after SMOTE of response variables for ACR and MCR outcomes. SMOTE, Synthetic Minority Over-Sampling Technique. It’s a good and powerful way to handle imbalanced data, and it was conducted under the parameters of *k* = 5, C.perc = “balance”, dist = “Overlap”. **(A)** ACR outcomes (before SMOTE); **(B)** MCR outcomes (before SMOTE); **(C)** ACR outcomes (after SMOTE); and **(D)** MCR outcomes (after SMOTE).

Imbalanced datasets are not unusual in medical research, because the number of non-patients is extremely larger than that of patients, which serves as an obstacle to predictive performance (15). The Synthetic Minority Over-Sampling Technique (SMOTE) is an oversampling technique that is an effective algorithm for handling imbalances between data classes (16). It uses k-neighbour synthesis to amplify minority classes to obtain a balanced data set (17) that exhibits good performance in areas such as network intrusion detection systems and disease detection. In this study, there is a serious imbalance in the response variables, ACR outcomes and MCR outcomes (Figures 2A, B).

### Three classifiers and evaluation parameters

Three models, i.e., RF (18, 19), Bagging (20, 21) and XGBoost (22, 23) were employed to make a classification of ACR outcomes and MCR outcomes, respectively. More detailed information about the models could be obtained in the Supplementary material.

Evaluation parameters include Accuracy (1), Specificity (2), Sensitivity (3) and Area under the receiver operating curve (AUC). When patients with kidney disease are classified as patients, the prediction is defined as True Positive (TP), and when a healthy person is classified as healthy, the prediction is defined as True Negative (TN). In addition, if healthy subjects are considered patients, the prediction is False Positive (FP); Similarly, False Negative (FN) if patients are considered healthy subjects. Accuracy is to evaluate how accurate the machine learning algorithms are to detect what it is supposed to measure. Specificity is the ability to correctly exclude those without renal conditions and Sensitivity is to correctly identify those with renal conditions.


(1) $$A\,c\,c\,u\,r\,a\,c\,y=\frac{\left(T\,N+T\,P\right)}{\left(T\,P+T\,N+\text{FP}+F\,N\right)}\times 100\%$$

(2) $$S\,p\,e\,c\,i\,f\,i\,c\,i\,t\,y=\frac{T\,N}{\left(T\,N+F\,P\right)}\times 100\%$$

(3) $$S\,e\,n\,s\,i\,t\,i\,v\,i\,t\,y=\frac{T\,P}{\left(T\,P+F\,N\right)}\times 100\%$$

### Statistical methods

Qualitative data are described as percentages (%), and quantitative data are expressed as mean standard deviation (M±SD) or median ± interquartile (P_25_, P_75_), as appropriate. Model construction: The datasets were divided into training set (80%) and testing set (20%). The former is used for model training, i.e., e, RF, Bagging, and XGBoost, while the latter is used for model performance evaluation. The comparisons between training set and testing set were conducted using *t*-test or non-parameter test for quantitative variables, and Chi-square test for qualitative variables. All analyses were implemented in R software (version 4.0.3).

## Results

### Baseline characteristics

A total of 12,330 rural residents participated in this study, of whom 5,230 were men and 7,100 were women aged 40–91 years. There were 1,587 (12.8%) cases with increased ACR and 1,456 (11.8%) cases with increased MCR, as shown in Table 1. Features in the training set and testing set are comparable for both ACR outcomes (except TG and sex) and MCR outcomes (*P* < 0.05), as shown in Tables 2, 3.

**TABLE 1:** Baseline characteristics of participants in this study.

Variables	Levels	Men (*N* = 5,230)	Women (*N* = 7,100)
Age	Median (IQR)	59.0 (52.0, 67.0)	57.0 (51.0, 65.0)
Education levels	≤Primary	1,394 (26.7%)	2,632 (37.1%)
	≤Junior	2,811 (53.7%)	3,470 (48.9%)
	≤Senior	761 (14.6%)	703 (9.9%)
	≥Bachelor	264 (5%)	295 (4.2%)
Annual income	<5K	1,563 (29.9%)	3,594 (50.6%)
	5–10K	1,396 (26.7%)	1,745 (24.6%)
	10–20K	641 (12.3%)	633 (8.9%)
	>20K	1,630 (31.2%)	1,128 (15.9%)
TG (mmol/L)	Median (IQR)	1.5 (1.1, 2.1)	1.6 (1.1, 2.2)
TC (mmol/L)	Median (IQR)	4.0 (3.5, 4.6)	4.6 (4.0, 5.2)
LDL (mmol/L)	Median (IQR)	2.1 (1.6, 2.6)	2.4 (1.9, 3.0)
HDL (mmol/L)	Median (IQR)	1.2 (1.0, 1.4)	1.3 (1.1, 1.5)
FPG (mmol/L)	Median (IQR)	4.6 (4.1, 5.1)	4.8 (4.4, 5.4)
GHB (mmol/L)	Median (IQR)	5.4 (5.0, 5.8)	5.4 (5.0, 5.8)
SBP (mmHg)	Median (IQR)	133.5 (123.0, 146.0)	134.0 (122.0, 148.5)
DBP (mmHg)	Median (IQR)	82.5 (77.5, 90.0)	81.0 (75.5, 88.5)
Hcy (mmol/L)	Median (IQR)	21.7 (15.5, 34.0)	16.6 (12.5, 23.7)
BMI	Underweight	80 (1.5%)	124 (1.7%)
	Normal	2,046 (39.1%)	2,828 (39.8%)
	Overweight	2,231 (42.7%)	3,017 (42.5%)
	Obesity	873 (16.7%)	1,131 (15.9%)
Smoking	No	2,415 (46.2%)	6,977 (98.3%)
	Yes	2,815 (53.8%)	123 (1.7%)
Alcohol consumption	Rarely	3,421 (65.4%)	7,024 (98.9%)
	Sometimes	1,549 (29.6%)	73 (1%)
	Always	260 (5%)	3 (0%)
Exercise	Regular	2,189 (41.9%)	2,952 (41.6%)
	None or a little	3,041 (58.1%)	4,148 (58.4%)
Salt consumption	Light	1,242 (23.7%)	2,002 (28.2%)
	Moderate	3,157 (60.4%)	4,308 (60.7%)
	Salty	831 (15.9%)	790 (11.1%)
Diet	Vegetable	1,541 (29.5%)	2,590 (36.5%)
	Balanced	3,322 (63.5%)	4,308 (60.7%)
	Meat	367 (7%)	202 (2.8%)
ACR	Normal	4,745 (90.7%)	5,998 (84.5%)
	Increased	485 (9.3%)	1,102 (15.5%)
MCR	Normal	4,473 (85.5%)	6,401 (90.2%)
	Increased	757 (14.5%)	699 (9.8%)

**TABLE 2:** Comparisons of quantitative clinical indexes between training set and testing set.

	ACR outcomes			MCR outcomes		
	Training (*N* = 9,864)	Testing (*N* = 2,466)	*P*	Training (*N* = 9,864)	Testing (*N* = 2,466)	*P*
Age (y)	59.00 (52.00,67.00)	59.00 (53.00, 67.00)	0.199	61.00 (54.00, 68.00)	60.00 (54.00, 68.00)	0.147
TG (mmol/L)	1.63 (1.17, 2.27)	1.69 (1.20, 2.34)	0.02	1.61 (1.15, 2.23)	1.66 (1.15, 2.34)	0.052
TC (mmol/L)	4.42 (3.78, 5.08)	4.39 (3.77, 5.04)	0.273			
FPG (mmol/L)	4.80 (4.30, 5.50)	4.80 (4.30, 5.60)	0.333	4.80 (4.30, 5.50)	4.80 (4.30, 5.50)	0.611
GHB (mmol/L)	5.40 (5.00, 5.90)	5.40 (5.00, 6.00)	0.17	5.40 (5.00, 6.00)	5.40 (5.10, 6.00)	0.749
SBP (mmHg)	137.50 (126.00, 152.00)	137.50 (125.00, 152.00)	0.948	137.00 (126.00, 151.00)	137.00 (125.00, 151.00)	0.813
DBP (mmHg)	83.00 (77.50, 91.00)	83.000 (77.50, 91.00)	0.529	82.00 (77.00, 90.00)	82.50 (77.50, 90.50)	0.113
Hcy (mmol/L)	18.60 (13.60,27.90)	18.55 (13.60, 28.13)	0.611	19.30 (14.00, 29.60)	19.10 (13.80, 29.05)	0.087

**TABLE 3:** Comparisons of qualitative clinical indexes between training set and testing set.

Variables	ACR outcomes			MCR outcomes		
	Training (*N* = 9,864)	Testing (*N* = 2,466)	*P*	Training (*N* = 9,864)	Testing (*N* = 2,466)	*P*
**Education**						
≤Primary	3,591 (36.4)	860 (34.9)	0.093	3,749 (38.0)	924 (37.5)	0.554
≤Junior	4,929 (50.0)	1,288 (52.2)		4,829 (49.0)	1,193 (48.4)	
≤Senior	969 (9.8)	243 (9.9)		974 (9.9)	262 (10.6)	
≥Bachelor	375 (3.8)	75 (3.0)		312 (3.2)	87 (3.5)	
**Exercise**						
Regular	4,215 (42.7)	1,026 (41.6)	0.312			
None or a little	5,649 (57.3)	1,440 (58.4)				
**BMI**						
Underweight	158 (1.6)	34 (1.4)	0.784	165 (1.7)	41 (1.7)	0.748
Normal	3,426 (34.7)	867 (35.2)		3,753 (38.0)	951 (38.6)	
Overweight	4,225 (42.8)	1,041 (42.2)		4,220 (42.8)	1,026 (41.6)	
Obesity	2,055 (20.8)	524 (21.2)		1,726 (17.5)	447 (18.1)	
**Alcohol**						
Rarely	8,677 (88.0)	2,129 (86.3)	0.05	8,329 (84.4)	2,095 (85.0)	0.09
Sometimes	1,009 (10.2)	294 (11.9)		1,327 (13.5)	304 (12.3)	
Always	178 (1.8)	43 (1.7)		208 (2.1)	66 (2.7)	
**Smoking**						
No	7,849 (79.6)	1,943 (78.8)	0.391	7,267 (73.7)	1,824 (74.0)	0.744
Yes	2,015 (20.4)	523 (21.2)		2,597 (26.3)	641 (26.0)	
**Diet**						
Vegetable	3,273 (33.2)	831 (33.7)	0.798	3,448 (35.0)	835 (33.9)	0.569
Balanced	6,188 (62.7)	1,530 (62.0)		6,024 (61.1)	1,534 (62.2)	
Meat	403 (4.1)	105 (4.3)		392 (4.0)	96 (3.9)	
**Salt consumption**						
Light	2,606 (26.4)	678 (27.5)	0.173			
Moderate	6,065 (61.5)	1,467 (59.5)				
Salty	1,193 (12.1)	321 (13.0)				
**Sex**						
Male	3,523 (35.7)	934 (37.9)	0.046	4,551 (46.1)	1,124 (45.6)	0.631
Female	6,341 (64.3)	1,532 (62.1)		5,313 (53.9)	1,341 (54.4)	

### Feature selection and results of SMOTE

**FIGURE 3:**
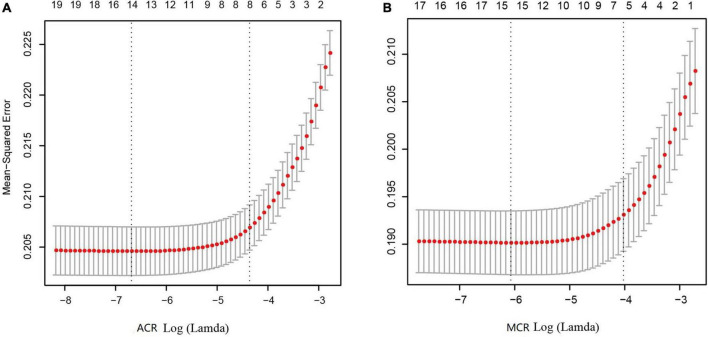
Results of feature selection using LASSO. When Lamda is minimum, corresponding features were taken into model construction, that is, 14 features for ACR outcomes **(A)** and 15 feature for MCR outcomes **(B)**.

As shown in Figures 3A, B, after LASSO feature selection, 14 and 15 explanatory variables remained in the two datasets, respectively. Dataset with ACR outcomes as the response variable excluded six variables of annual income, residence, LDL, HDL, smoking, and exercise; while dataset with MCR outcomes as the response variable excluded five variables of TC, LDL, HDL, exercise, and salt consumption. As shown in Figures 2C, D, after SMOTE resampling, the number of patients and normal ones were 6,165, 6,165 for ACR outcomes, and 6,165, 6,164 for MCR outcomes, respectively.

### Model performance

When constructing models for ACR outcomes, the number of increased ACR and normal ACR in the training set were both 4,932, and 1,233 in the testing set, respectively. When constructing model for MCR, the number of increased MCR and normal MCR in the training set were 4,973 and 4,891, respectively, and 1,273 and 1,192 in the testing set. The Accuracy, Sensitivity and Specificity of Bagging, RF and XGBoost reached over 99.00% in the training sets, and the AUC reached 0.99, as shown in Table 4. In the testing sets, XGBoost performed best, with Accuracy, Specificity and Sensitivity standing at 80.17, 77.05, and 83.29% for ACR outcomes and 82.27, 82.91, and 81.71% for MCR outcomes, and the AUC standing at 0.90. The performance of Bagging and RF is similar, as shown in Table 5.

**TABLE 4:** Performance evaluation of the three classifiers on the training set (ACR/MCR outcomes).

Model	Accuracy (%)	Sensitivity (%)	Specificity (%)	AUC
Bagging	99.91/99.91	99.96/99.90	99.96/99.92	0.99/0.99
RF	99.90/99.89	99.96/99.90	99.84/99.88	0.99/0.99
XGBoost	99.89/99.59	99.96/99.82	99.82/99.38	0.99/0.99

**TABLE 5:** Performance evaluation of the three classifiers on the testing set (ACR/MCR outcomes).

Model	Accuracy (%)	Specificity (%)	Sensitivity (%)	AUC
Bagging	78.30/80.37	74.05/82.36	82.56/78.77	0.87/0.88
RF	78.14/80.20	74.29/81.88	82.00/78.84	0.87/0.89
XGBoost	80.17/82.27	77.05/82.91	83.29/81.71	0.89/0.90

### Feature importances

**FIGURE 4:**
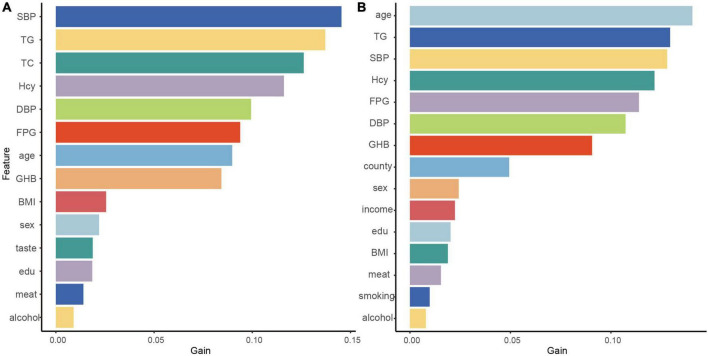
Contributions of explanatory variables to the XGBoost algorithm. The “Gain” means the relative contribution of the corresponding feature to the model calculated by taking the contribution of each feature to each tree in the model. The high value of this metric compared to other characteristics means that it is more important for generating predictions. Therefore, a larger value indicates that the variable is more important; ACR outcomes **(A)** and MCR outcomes **(B)**.

Since XGBoost performs best in the classification, we indicated the contribution of the explanatory variables to the model by Gain, and the larger the Gain, the more important the variables were for the XGBoost model. The five variables that contributed most to the classification of ACR outcomes in the XGBoost model represented SBP, TG, TC, and Hcy, DBP. The five variables that were most important for the classification of MCR outcomes constituted age, TG, SBP, Hcy and FPG (Figure 4).

## Discussion

To our knowledge, this study was the first one to employ machine learning algorithms in conjunction with existing indicators readily available in rural areas to construct an early warning model for CKD. In constructing the model, demographic information, physical examination and blood biochemical were taken as the explanatory variables; ACR (7) and MCR (24), early CKD screening parameters, collected and calculated from urine samples were taken as response variables. This study suggested that machine learning approaches show good performance in achieving early CKD-aided screening, as reflected by excellent Accuracy, Sensitivity, Specificity and AUC.

In 2012, Logistic Regression (LR) was employed for CKD prediction, but the model is accompanied by some defects (2), one of which concerns its sensitivity to multicollinearity. The second one relates to maximum likelihood estimation, unable to fit the true distribution of the data well. Recently, data-driven algorithms pick up pace, boasting great potential in cardiovascular diseases (25), tumors (26), immune diseases (27), and neurological diseases (28). Also, its application in renal diseases is increasing, ranging from acute kidney injury prediction (29) to kidney transplantation outcome prediction (30), interstitial fibrosis, and tubular atrophy detection (31).

In our previous work (32), we also employed LR, RF and Naive Bayes algorithms to make a classification of glomerular injury and tubular injury with the same population. The results suggested that RF performs best and could be employed as a novel auxiliary diagnostic approach for glomerular injury and tubular injury. To further compare the performance of other well-established algorithms, we, in this study, employed RF, Bagging and XGBoost to construct a warning model for CKD targeted at poverty-stricken areas with easily accessible parameters.

This study demonstrates that XGBoost represents the best classifier because the algorithm is a serial integrated learning algorithm based on Gradient Boosting Decision Tree that builds boosting trees in parallel by dependency generation (23). The objective function is improved by adding a regular term to the original function, thus reducing overfitting and speeding up convergence (33). Its extraordinary classification power in other diseases has also been demonstrated (34, 35). Besides, the five explanatory variables with the greatest output weight of XGBoost classifier for ACR outcomes represented SBP, TG, TC, and Hcy, DBP; and the five explanatory variables for MCR outcomes constituted age, TG, SBP, Hcy and FPG.

In hypertension, the early glomerular filtration rate could remain normal, but when arterial pressure is constantly rising, exceeding the kidney’s ability to self-regulate, it leads to glomerular hyperperfusion, which leads to damage to the visceral epithelial cells of the renal tubules, increasing the permeability of the glomerular basement membrane and thus causing proteinuria (36), while leading to glomerular duct wall hardening thickening, lumen stenosis, resulting in renal parenchymal ischemia, which eventually leads to glomerulosclerosis. Hyperhomocysteinemia is an important player involved in the progression of end-stage renal disease, acting directly on glomerular cells, inducing glomerular dysfunction and tubular fibrosis (37). Renal dyslipidemia is characterized by the accumulation of TG, which accelerates damage to the glomerular and tubular interstitials (38). Hyperglycemia levels not only enhance oxidative stress and hemodynamic factors such as activation of the renin-angiotensin-aldosterone system and impaired self-regulation due to systemic hypertension, but also increase the load of glucose delivered to the proximal tubules, triggering maladaptive hypertrophy (39) and hyperplasia of cortical tubules, and upregulation of glucose transport (40), and activation of globular feedback, leading to hyperfiltration of glomeruli and tubular fibrosis.

We think our early warning model is practical and down-to-earth in rural China. As the largest developing country confronted with underdeveloped healthcare systems and aging population (41), China is struggling to address the issue of health care coverage in rural areas. Despite the tremendous achievements in health care in rural China over the past 30 years, the problem of “difficult and expensive access to health care” still exists in the countryside. Promoting the establishment of a digital healthcare system in rural areas can greatly enhance the efficiency and accuracy of the public service system. Our study involves 12,330 participants from rural areas and how to better use such large-scale collected data to make a warning model for CKD is of great significance. Rather than considering these available indicators in isolation, holistically exploring their full potential, and seeking to explore a warning model for rural areas is a thing of great significance. Thus, early interventions tailored to CKD in improvised areas could be made in advance to lower its progression.

Some limitations also stand out in this manuscript. First, this study was based on a cross-sectional survey, and we did not conduct a follow-up for patients with proteinuria. Second, we constructed the models with data from Shanxi Province, and there is no other data for verification. Our next step is to collect more data from other regions to test the models’ generalization. Additionally, cost-effectiveness was initially considered in this study, and other indicators reflecting CKD, such as blood creatinine, were not collected, which is our next focus. Besides, we did not collect a detailed history of smoking, alcohol consumption and dietary intake. More detailed information would allow for a more accurate and valid model.

In short, CKD has emerged as a global public health issue and its early diagnosis is of great importance. In rural China where primary health care service system and health education remain to be improved and strengthened, how to construct a warning model for CKD targeted at poverty-stricken areas is of great necessity and significance. In this study, we proposed a warning model using the available and accessible demographical, blood biochemical and lifestyle data in conjunction with machine learning approaches for rural areas. This model offers a leg-up for early CKD-assisted diagnosis in rural areas, which facilitates tailoring precise management and therapy for patients, thus, improving their quality of life and slowing the mortality rate.

## Data availability statement

Data supporting the conclusions in this manuscript would be made available from the corresponding author upon reasonable request.

## Ethics statement

The studies involving human participants were reviewed and approved by Ethics Committee of Shanxi Provincial People’s Hospital. The patients/participants provided their written informed consent to participate in this study.

## Author contributions

WS and YLu were responsible for the data analysis and the writing of the manuscript. LQ and JQ helped polish the manuscript. AL, YZ, and YLi gave precious advice on the statistical methods. RL and XZ were responsible for the conception and design of the research. All authors read and approved the final draft.
