# Supplementary material for: Machine learning-based warning model for chronic kidney disease in individuals over 40 years old in underprivileged areas, Shanxi Province
Source: Front Med (Lausanne). 2023 Jan 9;9:930541. doi: 10.3389/fmed.2022.930541 (PMC9868668; doi:10.3389/fmed.2022.930541)
Supplement: Supplementary file 1 [file Table_1.DOCX]

# supplemental material--Four different classifiers

Logistic Regression (LR) (1, 2) represents one of generalized linear models. It assumes that the dependent variable y follows a Bernoulli distribution, while linear regression assumes that the dependent variable y follows a Gaussian distribution. Therefore, it has many similarities with linear regression, and the logistic regression algorithm is a linear regression if the Sigmoid mapping function is removed. It can be said that logistic regression is theoretically supported by linear regression, but logistic regression introduces nonlinear factors through the Sigmoid function, so it can easily handle classification problems.

eXtreme Gradient Boosting (XGBoost) is an improved algorithm based on gradient boosted decision trees (GBDT), which combines individual learners and can effectively establish the parallel operation of boosting trees by generating dependencies(3). It’s widely used in classification and regression for its fast operation, high efficiency, and high generalization ability. The algorithm improves the objective function by adding a regular term to the original function, thus reducing the possibility of overfitting and speeding up convergence(4).

Random Forest (RF) integrates multiple decision trees by “bagging”(5). A forest is built randomly under decision trees, many of which are constructed from the training set, and no correlations exist between the trees. A new sample is taken into the previously obtained forest if input, and the decision trees within the forest would judge the sample separately. The classification of the sample would be subject to the results of the trees. The model enjoys good accuracy and generalization for its good performance by integration of multiple weak classifiers or by voting(6).

Bagging (7, 8) is an integrated algorithm that reduces the generalization error by combining randomly generated training sets. The main idea is to train several different models separately and then let all models vote on the output of the test samples. The method reduces the variance of the base estimator (e.g., decision tree) by introducing randomness in the process of building the model. In most cases, the bagging method provides a very simple way to make improvements to a single model without modifying the algorithm behind it. Because bagging methods reduce overfitting, they usually perform well when used on strong classifiers and complex models.

1. Meurer WJ, Tolles J. Logistic Regression Diagnostics: Understanding How Well a Model Predicts Outcomes. Jama. 2017;317(10):1068-9.

2. LaValley MP. Logistic regression. Circulation. 2008;117(18):2395-9.

3. Davagdorj K, Pham VH, Theera-Umpon N, Ryu KH. XGBoost-Based Framework for Smoking-Induced Noncommunicable Disease Prediction. International journal of environmental research and public health. 2020;17(18).

4. Ogunleye A, Wang QG. XGBoost Model for Chronic Kidney Disease Diagnosis. IEEE/ACM transactions on computational biology and bioinformatics. 2020;17(6):2131-40.

5. Yang L, Wu H, Jin X, Zheng P, Hu S, Xu X, et al. Study of cardiovascular disease prediction model based on random forest in eastern China. Scientific reports. 2020;10(1):5245.

6. Dimitriadis SI, Liparas D. How random is the random forest? Random forest algorithm on the service of structural imaging biomarkers for Alzheimer's disease: from Alzheimer's disease neuroimaging initiative (ADNI) database. Neural regeneration research. 2018;13(6):962-70.

7. Lin E, Lin CH, Lane HY. Applying a bagging ensemble machine learning approach to predict functional outcome of schizophrenia with clinical symptoms and cognitive functions. Scientific reports. 2021;11(1):6922.

8. Lin E, Lin CH, Lane HY. Prediction of functional outcomes of schizophrenia with genetic biomarkers using a bagging ensemble machine learning method with feature selection. Scientific reports. 2021;11(1):10179.
